# Pituitary Macroadenoma and Severe Hypothyroidism: The Link between Brain Imaging and Thyroid Function

**DOI:** 10.1155/2021/2360855

**Published:** 2021-08-14

**Authors:** Silvia Ciancia, Silvia Cesari, Barbara Predieri, Sergio Bernasconi, Lorenzo Iughetti

**Affiliations:** ^1^Department of Medical and Surgical Sciences of the Mothers, Children and Adults, University of Modena and Reggio Emilia, Modena, Italy; ^2^Family Pediatrician, Parma, Italy; ^3^Microbiome Research Hub, University of Parma, Italy

## Abstract

In case of primary hypothyroidism, reactive pituitary hyperplasia can manifest as pituitary (pseudo) macroadenoma. We report the case of a 12-year-old boy who was evaluated for impaired growth velocity and increased body weight. Because of low insulin-like growth factor 1 levels and poor response to the growth hormone stimulation test, brain magnetic resonance imaging was performed and a pituitary macroadenoma was found. Treatment with levothyroxine was started, and thyroid function was evaluated approximately every 40 days to titrate the dosage. After few months of therapy, the size of the macroadenoma decreased and growth hormone secretion normalized. The pituitary returned to normal size in approximately 5 years. The boy went through puberty spontaneously and reached a normal adult height. In a patient affected by primary hypothyroidism, reactive pituitary hyperplasia can cause growth hormone deficiency; however, growth hormone secretion usually normalizes after starting levothyroxine treatment. Pituitary macroadenoma can be difficult to distinguish from severe pituitary hyperplasia; however, pituitary macroadenomas are rare in childhood, and our clinical case underlines how the hormonal evaluation is essential to achieve a correct diagnosis and prevent unnecessary surgery in a context of pituitary mass.

## 1. Introduction

Pituitary hyperplasia (PH) can be secondary to hypothyroidism (HT) as a consequence of high thyrotropin-releasing hormone (TRH) levels that stimulate both pituitary thyrotroph and lactotroph cells, leading to the enlargement of the pituitary gland and possibly hyperprolactinemia [1]. PH due to HT needs to be considered, especially in patients with severe and long-standing HT. In fact, as shown by Khawaja et al. [2], PH occurs in 70% of children with primary HT and thyroid-stimulating hormone (TSH) ≥50 mIU/L. In some patients, the pituitary gland can be significantly enlarged and, consequently, a macroadenoma may be included in the differential diagnosis, in particular when brain magnetic resonance imaging (MRI) is performed before thyroid tests. In childhood, pituitary macroadenomas are rare, accounting for less than 3% of all brain tumors, and are often difficult to diagnose. Their clinical manifestations include both endocrine and neurological signs and symptoms. The first ones are consequent to pituitary hormone dysfunction, while the neurological symptoms derive from the mass effect and can appear later than endocrine alterations and growth impairment [3]. Thus, in the suspicion of a pituitary macroadenoma, a careful endocrine evaluation is mandatory to avoid unnecessary and dangerous surgical procedures.

## 2. Case Description

**Figure 1:**
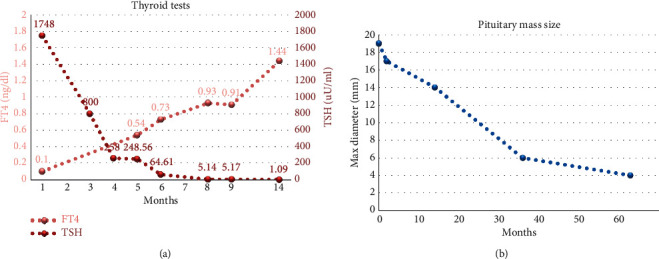
Biochemical and radiological follow-up. (a) TSH and FT4 values during levothyroxine treatment titration. (b) Reduction of the pituitary mass size during levothyroxine treatment, in a follow-up of around 5 years.

A 12-year-old boy was evaluated for statural growth deceleration and increased weight in a first-level hospital. In the previous year, the height decreased from the 50^th^ to the 25^th^ centile and the weight increased from the 50^th^ to the 75^th^ centile. Personal medical history was silent, and the family history was positive for autoimmune thyroiditis (his mother was affected). At the first clinical evaluation, auxological data were height 145.7 cm (−0.69 SDS), weight 39.05 kg, body mass index (BMI) 18.4 kg/m^2^ (0.29 SDS), Tanner's stages A1-P3-G3, and testicular volume 8 ml on both sides. The general physical examination was normal, except for mild facial swelling. Extremely increased value of TSH associated to low free thyroxine (FT4) and antithyroid antibodies led to the diagnosis of Hashimoto's disease: TSH 1748 *µ*U/ml (normal value 0.4–4.0), FT4 0.10 ng/dl (normal value 0.6–1.1), thyroid peroxidase antibody (TPOAb) > 1000 IU/ml (normal value < 6), and thyroglobulin antibody (TgAb) 587 IU/ml (normal value < 4). Ultrasound examination of the thyroid gland supported the diagnosis of autoimmune thyroiditis. Because of growth deceleration associated to insulin-like growth factor 1 (IGF-1) below the normal range for age, despite that the HT already explained the growth deceleration, a first stimulation test with arginine was performed to evaluate growth hormone (GH) secretion and an insufficient hormone response was demonstrated (GH peak 3.9 ng/ml). Bone age conformed to chronological age. The brain MRI showed an enlarged pituitary gland, with globular appearance and bulge of the upper profile, causing the stretching of the pituitary stalk. The mass measured 19 mm × 11 mm × 17 , and it reached the suprasellar cistern, imprinting the anterior portion of the optic chiasm; no cleavage plans with the cavernous sinus were evident bilaterally. After gadolinium administration, homogeneous enhancement was registered. The image was compatible with pituitary macroadenoma. At this time, after about 2.5 months, the patient was referred to our center for a second opinion. In view of high TSH levels associated with low levels of FT4 and GH, the suspicion of GH deficiency (GHD) secondary to pituitary hyperplasia associated to primary HT was raised. In the meanwhile, we started treatment with levothyroxine at 12.5 *µ*g/kg/day and thyroid function was evaluated approximately every 40 days to titrate the dosage (Figure 1(a)). After 3 months, when fT4 reached stable normal values, a second GH stimulation test demonstrated a normal GH peak (14 ng/ml). At this time, a first reduction of the pituitary enlargement was registered at MRI follow-up (Figure 1(b)). The boy was evaluated for the last time when he was 17 years and 7 months old: his final height was normal (−0.09 SDS), both weight and BMI were appropriate, and complete pubertal development was achieved. The last MRI showed a normal pituitary gland, with uniform signal, centered pituitary stalk, and normal optic chiasm.

## 3. Discussion

Primary hypothyroidism in childhood often occurs with unspecific symptoms and signs: among these, deceleration of height growth and gain of body weight can be the predominant clinical manifestations [4]. Due to the increased availability of MRI, brain imaging is more and more often performed at an early stage of the diagnostic workup for impaired growth. In case of severe primary hypothyroidism, reactive pituitary hyperplasia is not unusual, but because of the similarity between the imaging of pituitary macroadenoma and severe pituitary hyperplasia, the differential diagnosis can be challenging. In particular, if MRI is incorrectly performed before thyroid function tests, PH secondary to HT can be misdiagnosed with a macroadenoma. In fact, when the size of the pituitary gland is highly increased, growth deceleration can develop and might be incorrectly attributed to the compression of a pituitary adenoma on the pituitary stalk, resulting in decreased GH secretion.

Traditional diagnostic criteria for pituitary macroadenoma include homogeneous enlargement of the gland greater than 10 mm, with or without erosion of the sellar floor, and deviation of the stalk [5]. According to the more recent literature, macroadenomas are frequently asymmetric, the pituitary stalk can be deviated but only rarely thickened, the posterior pituitary bright spot is preserved, and the sellar floor can be eroded [6]. After gadolinium administration, pituitary hyperplasia reveals typically homogeneous enhancement on TI-W images while a macroadenoma may show either homogeneous or heterogeneous enhancement [7].

To discuss deeper the risk of misdiagnosis between pituitary macroadenoma and PH secondary to HT, we performed a literature research on the PubMed database using the combination of (primary hypothyroidism) AND (pituitary macroadenoma) AND (pediatric OR children). A total of 32 articles were provided, dated up to December 2020. Of these, three were excluded because the text was available only in Chinese, seventeen were excluded on the basis of the title and/or the abstract, and three were not included because the authors described a pituitary hyperplasia and not a pituitary adenoma (moreover, one of these case reports referred to a lady 29 years old, therefore outside the pediatric age). Of the nine case reports left, one was a letter to the editor written in response to one of the eight papers left that represent the final result of our selection (Table 1) [8–15].

Among the cases described, three patients underwent brain MRI as first exam because of neurological symptoms: one patient referred nausea and vomiting since ten months [8], the second patient presented with frontal headache, lethargy, and dizziness since two months [10], and the third patient complained of persistent occipital headache and growth arrest since three months [14]. In one of the two cases described by Young et al. [9], the MRI was performed before thyroid tests because the girl presented with galactorrhea and prolactin was increased [9]. In other patients, MRI was performed precociously for diminished growth velocity and excessive weight gain [9, 11] or for aspecific symptoms including headaches [12]. In all patients, a pituitary mass suggestive of macroadenoma was found. During the assessment of pituitary function, high levels of TSH associated with low levels of FT4 were detected and allowed the diagnosis of primary hypothyroidism. The pituitary mass was reinterpreted as pituitary hyperplasia, and the right treatment was started. On the other hand, brain MRI was also performed in some patients after hormonal workup for reduced GH levels [13, 15], as in the case we reported.

All cases we discussed show that, in the presence of a pituitary mass, it is essential to correlate imaging findings with hormonal evaluation, above all when brain MRI is performed for nonspecific neurological symptoms (such as headache) and, thus, before endocrine laboratory workup. In the presence of extremely high levels of TSH, a diagnosis of TSH-secreting adenoma should be excluded, but in this case, FT4 levels would be increased [12]. When high levels of TSH are associated with low levels of FT4, the finding of an increased pituitary gland on MRI imaging should be attributed firstly to PH secondary to HT. In this condition, the size of the pituitary will decrease after treatment with levothyroxine and unnecessary (and even dangerous) surgery is spared.

Moreover, it is conceivable that the extreme hypertrophy of thyrotroph cells leads to compression of the surrounding cells, causing mainly GHD and, as a consequence, growth deceleration. Usually, GH replacement therapy is not needed because impaired GH secretion often resolves after thyroid hormone replacement therapy [16, 17]. In any case, close monitoring is strongly recommended and persistence of GHD after long levothyroxine treatment has been described [12, 16].

## Figures and Tables

**Table 1:** Pituitary (pseudo) macroadenoma secondary to primary hypothyroidism: literature review.

Author and year	Age (yrs)	Gender	Symptoms and signs	Laboratory tests	Imaging findings (brain MRI)	Treatment	MRI follow-up
Ehirim et al., 1998 [8]	13	Male	Nausea and vomiting since 10 months	TSH 827 mIU/l (range, 0.5–5.0) FT4 <1 *µ*g/dl (range, 5–12)	Large homogeneously enhancing sellar and suprasellar pituitary mass, abutting the optic chiasm	Levothyroxine	Complete resolution of the pituitary mass one year after the start of thyroxine therapy

Young et al., 1999 [9]	14	Female	Galactorrhea, recurrent mild occipital headaches, and occasional visual disturbance (loss of peripheral vision) since 6 months	Prolactin 58 ng/ml (range, 3–30) TSH >100 mIU/l (range, 0.55–3.90) FT4 <2 mcg/dl (range, 4.4–12.2) TPOAb and TgAb +	Large pituitary mass compatible with a pituitary macroadenoma	Levothyroxine	Complete resolution of the pseudoadenoma (timing not available)
	6.75	Female	Pubic and axillary hair growth since 6 months, impaired statural growth, and excessive weight gain over the previous year	TSH >60 mIU/l (range, 0.46–4.98) FT4 2.1 mcg/dl (range, 5–12.5)	Sellar/supersellar mass of 17 × 17 mm, consistent with a pituitary adenoma	Levothyroxine	Complete resolution of the mass with normal pituitary and suprasellar anatomy (timing not available)

Hopper and Albanese, 2005 [10]	11	Female	Frontal headaches, lethargy, and dizziness since 2 months	TSH >1500 mIU/l (range, 0.4–4) FT4 0.3 pmol/l (range, 12–24) TPOAb 149 IU/ml (range, 0–109)	Greatly enlarged pituitary gland with homogeneous pituitary tissue impinging on the theoptic chiasm consistent with a pituitary macroadenoma	Levothyroxine	Reduction in the size of the pituitary gland to almost normal size, 5 months after presentation and start of treatment
Eom et al., 2008 [11]	9	Female	Impaired statural growth and excessive weight gain over the previous year	TSH 57.07 mIU/l (range, 0.2–5) FT4 0.01 ng/dl (range, 0.7–2)	Large intrasellar and suprasellar pituitary mass, homogeneous enhancing, convex upper contour, and a craniocaudal diameter of 20 mm. Upward extension, encroaching of suprasellar cisterna, superiorly displacing the optic chiasm	Levothyroxine	Complete resolution of the pituitary mass and partially empty sella, 4 months after starting the treatment

Simsek et al., 2009 [12]	14.5	Male	Short stature (−3.6 SDS), delayed puberty, headache, easy fatigability, extreme cold intolerance, and chronic constipation	TSH 334 mIU/l (range, 0.5–5) FT4 5.2 pmol/l (range, 9–26) TPOAb and TgAb (sublingual thyroid diagnosed on imaging)	Large pituitary mass with homogeneous enhancement, consistent with a pituitary macroadenoma.	Levothyroxine	Complete resolution of the enlarged pituitary, 6 months after levothyroxine replacement
	13	Female	Severe menorrhagia since 6 months, cold intolerance, marked fatigue, sluggishness, and difficulty in school	TSH 232 mIU/l (range, 0.5–5) FT4 2.7 pmol/l (range, 9–26) TPOAb 855 IU/ml (range, 0–35) TgAb 22 IU/ml (range, 0–40)	Homogeneously enhancing enlarged pituitary (height 11 mm) with suprasellar extension	Levothyroxine	Resolution of the pituitary enlargement, 6 months after starting the treatment

Cekmez et al., 2011 [13]	12	Female	Growth retardation and dry and lifeless hair	TSH >150 mIU/ml (range, 0.24–4) FT4 0.27 ng/dl (range, 0.58–1.64) TPOAb 31.2 IU/ml (range, 0–60)	Mass of 16 × 12 mm in the anterior lobe of the pituitary gland consistent with a macroadenoma, with slight extension into the right suprasellar cisterns	Levothyroxine	Reduction in the size of the lesion, 3 months after starting the treatment

Franceschi et al., 2011 [14]	10	Male	Occipital headache over the last three months and height growth arrest over the last to 2 years (decline from the 75–90^th^ centile to the 25^th^)	TSH 589 mU/l (range, 0.20–4.50) FT4 1.5 pmol/l (range, 12–22)	Pituitary mass isointense to gray matter, extended on the suprasellar cistern with mild compression of the optic chiasm. The pituitary stalk and posterior pituitary dislocated. After gadolinium, homogeneous enhanced	Levothyroxine	Resolution of the mass, 5 months after starting the treatment

Eklioglu et al., 2013 [15]	2.75	Female	Short stature (height −2.85 SDS)	TSH >100 mU/l (range, 0.6–5.5) FT4 0.62 ng/dl (range, 0.8–2.2)	Mass of 16 × 13 mm with homogeneous enhancement and extension to suprasellar cisterna, reported as macroadenoma	Levothyroxine	The lesion disappeared one year after starting the treatment

## Data Availability

All data generated or analyzed during this study are included in this published article. The data supporting this systematic review are from previously reported studies and datasets, which have been cited.

## Abbreviations:

PH: Pituitary hyperplasia
HT: Hypothyroidism
TRH: Thyrotropin-releasing hormone
TSH: Thyroid-stimulating hormone
FT4: Free thyroxine
MRI: Magnetic resonance imaging
IGF-1: Insulin-like growth factor 1
GH: Growth hormone
GHD: Growth hormone deficiency.

## Supplementary Materials

Supplementary Materials CARE checklist.
